# Effect of winter cold duration on spring phenology of the orange tip butterfly, *Anthocharis cardamines*

**DOI:** 10.1002/ece3.1773

**Published:** 2015-11-07

**Authors:** Sandra Stålhandske, Philipp Lehmann, Peter Pruisscher, Olof Leimar

**Affiliations:** ^1^ Department of ZoologyStockholm University106 91StockholmSweden

**Keywords:** Chill duration effects, diapause, insect phenology, local adaptation, post‐diapause development, post‐winter development, respirometry

## Abstract

The effect of spring temperature on spring phenology is well understood in a wide range of taxa. However, studies on how winter conditions may affect spring phenology are underrepresented. Previous work on *Anthocharis cardamines* (orange tip butterfly) has shown population‐specific reaction norms of spring development in relation to spring temperature and a speeding up of post‐winter development with longer winter durations. In this experiment, we examined the effects of a greater and ecologically relevant range of winter durations on post‐winter pupal development of *A. cardamines* of two populations from the United Kingdom and two from Sweden. By analyzing pupal weight loss and metabolic rate, we were able to separate the overall post‐winter pupal development into diapause duration and post‐diapause development. We found differences in the duration of cold needed to break diapause among populations, with the southern UK population requiring a shorter duration than the other populations. We also found that the overall post‐winter pupal development time, following removal from winter cold, was negatively related to cold duration, through a combined effect of cold duration on diapause duration and on post‐diapause development time. Longer cold durations also lead to higher population synchrony in hatching. For current winter durations in the field, the *A. cardamines* population of southern UK could have a reduced development rate and lower synchrony in emergence because of short winters. With future climate change, this might become an issue also for other populations. Differences in winter conditions in the field among these four populations are large enough to have driven local adaptation of characteristics controlling spring phenology in response to winter duration. The observed phenology of these populations depends on a combination of winter and spring temperatures; thus, both must be taken into account for accurate predictions of phenology.

## Introduction

**Figure 1:**
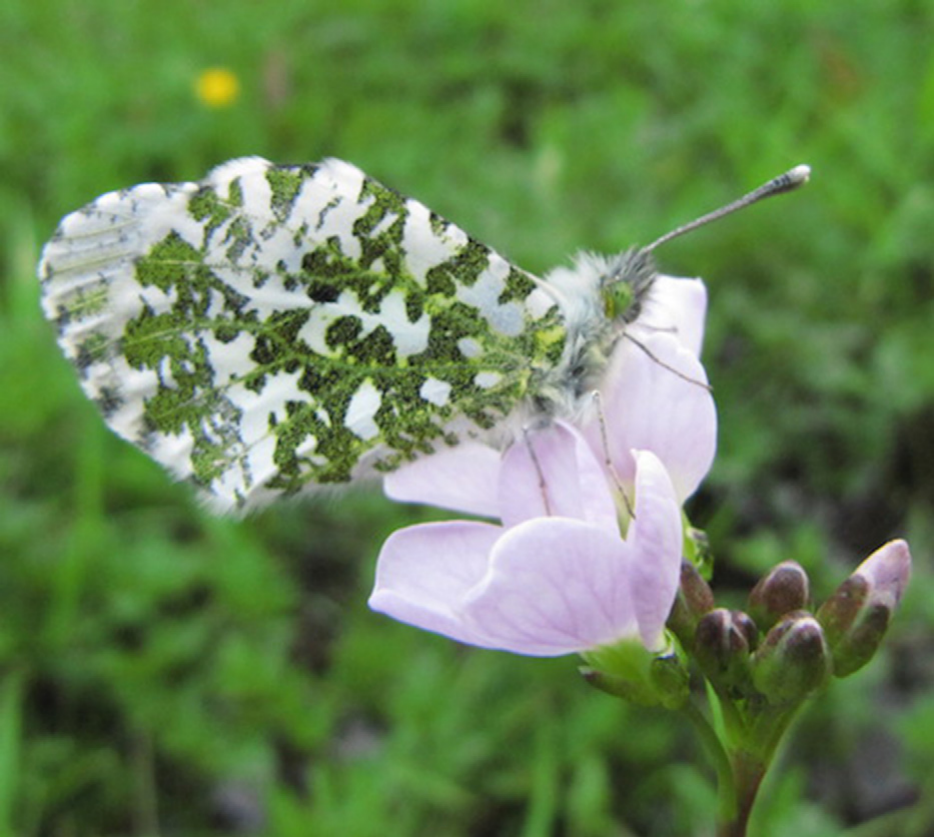
*Anthocharis cardamines* resting on one of its preferred host plant species *Cardamine pratensis*. Photographer: Sandra Stålhandske.

**Figure 2:**
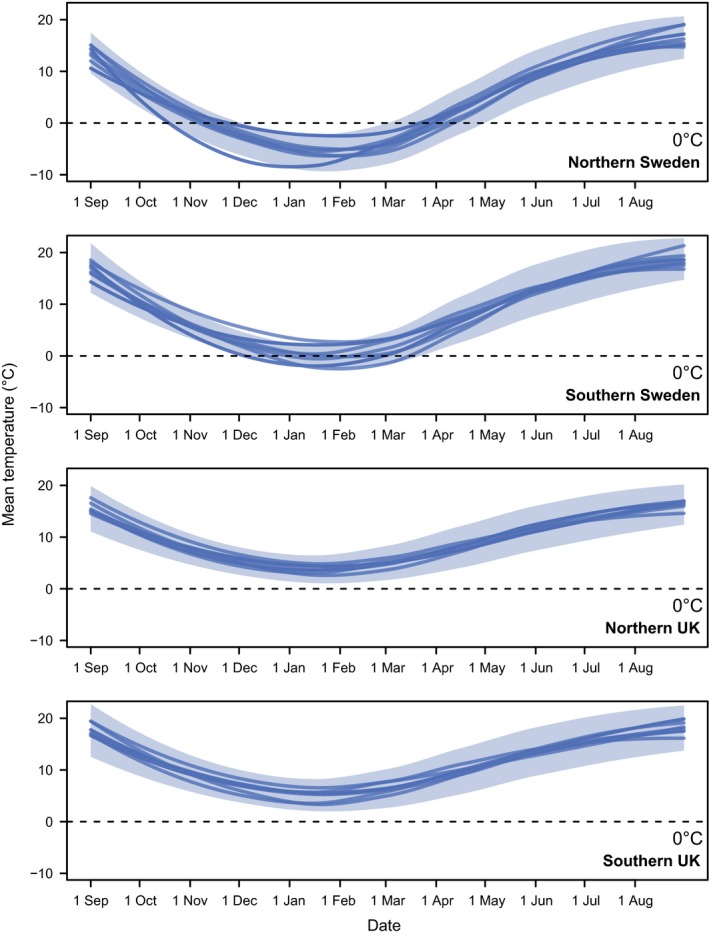
Temperature profiles for four localities in the United Kingdom and Sweden. Solid lines are mean temperatures for the years 2000–2009 (from the E‐OBS database). The transparent areas show the ranges of the average minimum and maximum temperatures over the same time period.

The scientific interest in phenology has increased concurrently with an awareness of climate change. Much research effort has been invested into predicting and understanding phenology of species at temperate latitudes (reviewed by Walther et al. 2002; meta‐analysis by Parmesan & Yohe 2003; Parmesan 2007). A majority of the reported phenological changes deals with spring phenology, which is often studied in relation to spring temperature (Badeck et al. 2004; Menzel et al. 2006; Polgar and Primack 2011). However, also winter temperature and duration can affect spring phenology in both plants (Murray et al. 1989; Cook et al. 2012; Laube et al. 2014) and insects (Collier and Finch 1983; Bosch and Kemp 2003; Chuche and Thiéry 2009; Xiao et al. 2013; Williams et al. 2015b). Here, we study the effect of winter duration on aspects of post‐winter development of four latitudinally separate populations of *Anthocharis cardamines* (orange tip butterfly) (Fig. 1). Two populations are from Sweden and two are from the United Kingdom. These populations experience different winter conditions and durations in the field, naturally varying from about 1 month in the UK to up to 4 or 5 months of winter in Sweden (Fig. 2). Our experimental setup allows us to study adaptations in the fine‐tuning of spring phenology to different winter conditions. We are also able to partition the overall timing effects of cold duration into diapause duration and post‐diapause development.

### Effects of chilling duration

Vernalization effects, or chilling requirements, are well studied in plants. For many plant species, there is a dynamic relationship between winter temperature and spring phenology (Erez et al. 1979; Porter and Gawith 1999; Cook et al. 2012). Compared to many agricultural vernalization models and the level of understanding for plant model species (e.g., Song et al. 2012), the influence of winter temperatures on insect phenology has been relatively little explored. Effects of winter conditions on insect life history have mainly focused on thermal stress tolerance and energetics (Addo‐Bediako et al. 2000; Marshall and Sinclair 2015; Williams et al. 2015a). Research on the effects of cold duration has been studied to a lesser degree, focussing on the amount or duration of cold needed to break diapause (Collier and Finch 1983; Bosch and Kemp 2003; Chuche and Thiéry 2009). There are also some studies on insects showing that developmental trajectories are indeed affected by cold duration, which may serve to synchronize local populations (Xiao et al. 2013; Chen et al. 2014).

### Diapause and post‐diapause development

Diapause, a state of reduced developmental activity and metabolic rate, is near ubiquitous in insects at high latitudes and altitudes (Tauber et al. 1986). Diapause is a preprogrammed state during which the insect is typically unresponsive to external cues for initiation of development (Tauber and Tauber 1976; Koštál 2006). Diapause termination is difficult to study as there is no easily discernable physiological change of the transition from diapause to post‐diapause quiescence (Wadsworth et al. 2013). In this latter stage, the individual remains in a state of reduced metabolic and developmental activity, but is again responsive to stimuli from the external environment (Tauber and Tauber 1976). Development can then resume if external conditions allow, a phase termed post‐diapause development (Tauber and Tauber 1976). The timing of the transition from diapause to post‐diapause quiescence as well as when the resumption of development occurs is of great interest for understanding phenology and studying phenological change.

Post‐diapause development rate in insect eggs as well as larvae and pupae is dependent on both temperature and physiological age, or time in development (Regniere 1990; Gray 2009; Stålhandske et al. 2014). There is an inherent increase in development rate nearer to emergence as well as an increased sensitivity to temperature closer to hatching (Gray 2009). The post‐winter development of *A. cardamines* has previously been shown to have two distinct phases of linear weight loss (Stålhandske et al. 2014). The second phase shows a near four‐fold increase in the rate of weight loss compared to the first phase (Stålhandske et al. 2014). This ramping up of development may benefit insects, such that conspecifics in a local area are more likely to emerge at the same time, thereby increasing the chance of finding a mate. Also because of weather variation, such ramping up can increase the chances of emerging during a high pressure. Emerging in good weather is important for butterflies for finding mates and host plants.

In this study, we examine the effect of winter duration on the characteristics of post‐winter development of *A. cardamines*. Firstly, we look at the effect of cold duration on overall post‐winter development time. Secondly, we partition this effect between effects on diapause duration and on post‐diapause development, using weight loss data. Finally, in order to corroborate our methods and assumptions, on a subset of individuals, we measure the CO_2_ (carbon dioxide) production, which is a trait more closely and mechanistically linked to metabolic rate than weight loss. We also measure the proportion of water content in the different developmental stages, to more accurately judge the relationship between metabolic rate and weight loss.

## Methods

### Study species

*Anthocharis cardamines* is a univoltine butterfly species that has an obligate pupal diapause and emerges as an adult in the spring across its European range (Courtney 1980). It is oligophagous and oviposits on young inflorescences of several species of Brassicaceae (Wiklund and Åhrberg 1978). The phenological state of a plant strongly influences oviposition choices, and larval survival differs among host plant species (Wiklund and Åhrberg 1978; Courtney 1981). The protein‐rich seedpods are the main food source for the larvae (Wiklund and Åhrberg 1978).

### Laboratory rearing

Eggs from laboratory‐reared butterflies from two latitudinally separate localities in the UK (Bournemouth: Lat 50.72N, Lon 1.88W, *N* = 48 eggs, 15 females, 20 males, 13 unhatched pupae; Durham: Lat 54.68N, Lon 1.57W, *N* = 41 eggs, 10 females, 17 males, 14 unhatched pupae) and two latitudinally separate localities in Sweden (Skåne: Lat 55.49N, Lon 14.05E, *N* = 26, 10 females, six males, 10 unhatched pupae; Ångermanland: Lat 63.03N, Lon 18.19E, *N* = 64, 20 females, 28 males, 16 unhatched pupae) were reared to pupation in a common garden setting (17°C and 12L:12D) with ad libitum access to garlic mustard (*Alliaria petiolata*) in the late spring and early summer of 2013. The larvae were kept at low density (three in a 500‐mL rearing cup). After pupation, individuals were kept singly in plastic containers. All pupae were put into cold treatment (2.4 ± 0.7°C and 0L:24D) in the late autumn of 2013.

### Experimental procedure

Equal numbers of pupae from each of the four populations were taken out to a warm treatment (16.2 ± 0.3°C and 18L:6D) after 30 days, 60 days, 90 days, and 120 days in cold (Table S1). The post‐winter development was monitored by weighing pupae every 48 h on a Precisa 205A SCS electrobalance (±0.0002 g). Weights were recorded in units of 0.0001 g. The rate of weight loss was used as a rough proxy for the rate of development (Forsberg and Wiklund 1988; Posledovich et al. 2014; Stålhandske et al. 2014). The number of days from introduction to the warm temperature treatment until hatching was recorded, with the day taken out of the cold considered as day zero. The sex of the individuals was determined by adult wing morphology.

### Respirometry

To investigate the release of metabolic suppression in pupae transitioning from diapause to post‐diapause development and to clarify the ontogenetic trajectory of the developing pupae, CO_2_ (carbon dioxide) production (reflecting metabolic rate) was measured from a subset of pupae from each cold treatment group. CO_2_ production was measured overnight in eight individuals from the 30‐day cold treatment group, five individuals from the 60‐day cold treatment group, and one individual from the 90‐day cold treatment group. CO_2_ production was measured using a Sable Systems (Las Vegas, NV) respirometry system set to absolute mode. We used a stop‐flow setup with eight measurement chambers. Each chamber was filled with ambient air and closed for 45 min, after which the chamber was flushed for 5 min at a flow rate of 50 mL per min using an SS4 (Sable Systems) subsampler pump. Chambers were cycled with a MUX multiplexer (Sable Systems). Upon leaving the measurement chamber, air water content was measured with a RH‐300 meter (Sable Systems). Water was subsequently scrubbed with a 20‐mL column containing magnesium perchlorate. CO_2_ content in the air stream was measured using a Li‐7000 CO_2_ analyzer (LiCor, Lincoln, NE).

For an additional five individuals who had experienced 150 days in cold, CO_2_ production was measured overnight (approximately 16:00–9:00) every night until hatching with a differential respirometry setup. Again a stop‐flow system was used, but cycles were now 25 min long. Two separate lines of air scrubbed of H_2_O and CO_2_ using drierite (WA Hammond Drierite) and ascarite (Acros Organics) scrubbers, respectively, were pushed at a steady rate of 70 mL/min using a SS4 subsampler. The sample line air was humidified to 0.7 kPa with a water bubbler (which was controlled with RH‐300). The air then went into the MUX multiplexer and through each animal chamber, after which it entered the sample line of the Li‐7000 CO_2_ analyzer. The second line proceeded the same way, mimicking the exact length of the sample line, entering the reference line of the Li‐7000. Differential CO_2_ was calculated by subtracting the output of the reference line from the output of the sample line. For all measurements, sampling rate was 1 Hz. The raw output was baseline corrected, fractioned, and multiplied with flow rate (Lighton 2008). Finally, the output was mass corrected with pupal weight to yield mL CO_2_/min/g.

### Water loss

To investigate how post‐diapause weight loss is related to a loss of water, the proportion of weight that is water was determined in pupae in three different stages of post‐winter development. Water content was measured gravimetrically. Pupae were weighed (fresh weight), then punctured in 10 locations along the ventral side, dried individually in cups at 55°C for 72 h, and re‐weighed (dry weight). The water content was calculated by dividing dry weight with fresh weight. First, eight pupae in the post‐diapause quiescence stage were taken immediately from cold conditions. Second, based on the two‐phase post‐winter development previously reported (Stålhandske et al. 2014), a measurement was performed on seven other pupae in the first phase of post‐winter development (following 3 days at 17°C). Third, a measurement was performed on four further pupae in the second phase (following 9 days at 17°C).

### Data analyses

All statistical tests were performed using the statistical software R, version 3.1.2 (R Core Team 2014). For parametric statistics, model selection was performed by starting with a full model and stepwise removing nonsignificant interactions and explanatory variables. For linear models, the initial pupal weight was used as a covariate to exclude possible effects on development of differences in pupal size among localities. The covariate was removed in the final model if nonsignificant. For all parametric models, a transformation of the response variable was performed if needed to meet the assumptions of the test. For all final models, there was normality of residuals and homogeneity of variances.

### Estimation of diapause termination

The individuals from the 30 and 60 days cold treatments that did not hatch after 150 days of warm treatment were known to remain in diapause, and were found to have a low but steady rate of weight loss after being removed from cold (mean ± SD: 0.000084 ± 0.000042 g/day). Other individuals, with similarly low rates of weight loss after removal from cold, were assumed to be in diapause at the start of the warm treatment. In order to determine when these individuals left diapause, a limit given by the mean plus two standard deviations of the weight loss of the individuals known to be in diapause was applied. For each day, the rate of weight loss over the following three weight measurements (6 days) was estimated, following introduction to the warm treatment. The day when this rate of weight loss of an individual exceeded the limit, it was said to have left diapause and to have initiated development.

### Post‐winter and post‐diapause development times

We define the post‐winter development time *t*_*P*_ as the time in days from removal of a pupa from cold to the day of hatching as an adult, with the day of removal counted as zero. Using the above criterion for diapause termination, we refer to the time from removal from cold to diapause termination as *t*_0_, with *t*_0_ = 0 if a pupa had terminated diapause already during the cold period. The post‐diapause development time, *t*_*D*_, is the time from initiation of development until hatching as adult, so that *t*_*P*_ = *t*_0_ + *t*_*D*_. We define corresponding developmental rates as *r*_*P*_ = 1/*t*_*P*_ and *r*_*D*_ = 1/*t*_*D*_.

Differences among the populations in the propensity to hatch after the cold treatment were investigated using a chi‐square test. A linear model was used to analyze overall post‐winter development time *t*_*P*_. The proportion of immediately developing individuals in the 60, 90, and 120 days cold treatment was investigated using logistic regression. The shortest cold duration treatment was not included in this analysis, as no individuals started developing after 30 days in cold. The time *t*_0_ to development initiation after removal from cold of the individuals that initially were in diapause was analyzed using a linear model. For the analysis of the post‐diapause development rate *r*_*D*_, males and females were analyzed separately, so that the assumptions of the tests were met.

### Annual temperature profiles in Sweden and the UK

The daily mean temperature profiles of the Swedish and British localities were estimated over the years 2001–2009 using the freely available gridded daily temperature data from E‐OBS version 11.0 (Haylock et al. 2008), with a 0.5° longitude by 0.5° latitude grid. The daily minimum and maximum temperature profiles were also computed over the years 2001–2009.

## Results

### Effects of cold on diapause duration and post‐diapause development

Thirty days of cold conditions were not enough for any of the populations to break diapause. After 150 days in warm conditions following the 30‐day cold treatment, none of the pupae had hatched (*n* = 43). After 60 days in cold, a higher proportion of *A. cardamines* from the southern UK (92%) and northern Sweden (94%) hatched compared to individuals from northern UK (63%) and southern Sweden (43%), and this is a statistically significant difference ($\chi_{\mathrm{df}=3}^{2}$ = 10.5, *P *=* *0.015). No females from southern Sweden hatched after 60 days in cold.

**Figure 3:**
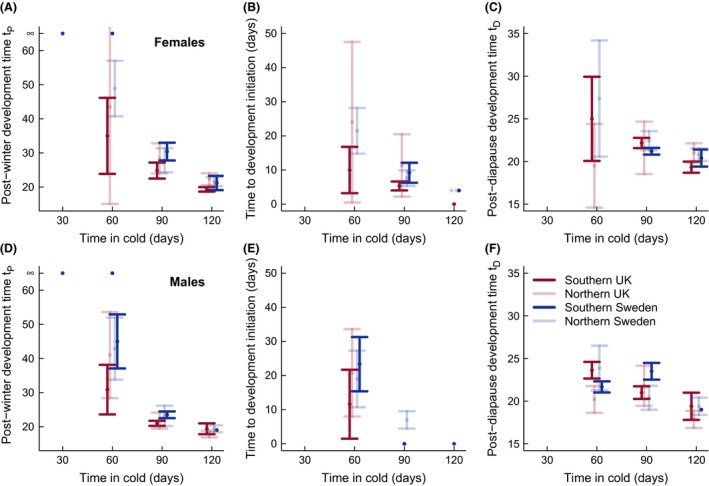
Effect of cold duration on post‐winter development. Error bars show the 95% confidence interval of the mean. (A, D) Effect of cold duration on development time from removal from winter conditions to hatching for the four populations. (B, E) Effect of cold duration on the time to development initiation following introduction to spring conditions. (C, F) Effect of cold duration on the time in post‐diapause development.

The overall post‐winter development time (*t*_*P*_) depended on duration of cold, sex, and locality (Table 1, Fig. 3A and D), in agreement with previous results from Swedish populations that included an overlapping winter duration treatment of 90 days (Posledovich et al. 2015). The time showed a steep decline with increasing duration of cold; there was approximately a halving of the time spent in post‐winter development from 60 days to 120 days in cold. For the shorter cold durations, the post‐winter development time of the UK pupae was shorter than that of the Swedish pupae, but for longer winter durations, the times were fairly similar (Fig. 3A and D).

**Table 1:** Statistical analysis of the effects of cold duration and locality on the post‐winter development time *t*_*P*_ (*t*_*P*_^−1.5^), see Figure 3A and D

Effect	df	*F*	*P*
Cold days	2	184.7	<**0.001**
Sex	1	35.9	<**0.001**
Locality	3	6.5	<**0.001**

Statistically significant values are shown in bold.

We split the overall post‐winter development time *t*_*P*_ into the time *t*_0_ taken to initiate development following removal from cold (i.e., time in diapause) and the post‐diapause development time *t*_*D*_. Of the pupae that hatched, the proportion of individuals that initiated development immediately after having been moved to warm conditions differed with cold duration and among populations (Tables 2, 3), and the proportion increased with the amount of cold experienced (Table 2). The southern UK population had the highest proportion of individuals that developed immediately (71% of the 60‐, 90‐, and 120‐day cold treatments). There was also a statistically significant effect of cold duration on the time to development initiation after removal from cold, as well as population differences in this time (Table 3, Fig. 3B and E). Individuals from the southern UK population started developing in a few days after 60 days in cold, whereas for the other populations, the time in warm conditions before development started was around 20 days (Fig. 3B and E). Following 120 days in cold, nearly all individuals (93%) developed immediately after being taken out of the cold.

**Table 2:** Proportion of individuals that were in diapause immediately after being taken out of the cold treatment for the different cold durations and localities. Only individuals that hatched as adults during 150 days of warm conditions are included

Cold duration	N. Sweden		S. UK		N. UK		S. Sweden	
	Females	Males	Females	Males	Females	Males	Females	Males
60	1	1	0.67	0.63	1	1	NA	1
90	0.71	0.40	0.50	0	0.60	0	1	0
120	0.20	0	0	0	0.33	0	0.20	0

**Table 3:** Model summaries of the effects of cold duration, locality, and sex on post‐winter development of *Anthocharis cardamines*. 1. Logistic regression of the proportion of individuals initially in diapause after removal from cold (see Table 1). 2. Linear model of the time to development initiation after removal from cold (see Fig. 3B and E)

Effect	1. Proportion of individuals initially in diapause			2. Time to development initiation (log(days))		
	df	LR *χ* ^2^	*P*	df	*F*	*P*
Cold days	1	80.8	<**0.001**	1	46.9	<**0.001**
Locality	3	15.3	**0.002**	3	5.72	**0.002**
Sex	1	17.0	<**0.001**			
Cold days: sex	1	3.5	0.061			

Locality, sex and time in cold affected the post‐diapause development rate *r*_*D*_ (Table 4). The cold duration was the most important factor explaining variation in the post‐diapause development time (Fig. 3C and F). The Swedish and UK individuals had similar development times for the cold durations used in this experiment (Fig. 3C and F). From previous results (Stålhandske et al. 2014), after 150 days of cold conditions, Swedish individuals have a higher post‐diapause development rate than UK individuals.

**Table 4:** Model summaries for effect of cold duration on post‐diapause development for males and females (see Fig. 3C and F)

Effect	Males (*r*_*D*_)			Females (*r*_*D*_^*3*^)		
	df	*F*	*P*	df	*F*	*P*
Cold days	2	19.3	<**0.001**	2	6.2	**0.004**
Locality	3	2.4	0.076	3	2.1	0.12
Initial weight	1	2.3	0.13	1	1.1	0.29
Locality: initial weight	3	3.2	**0.031**	3	4.0	**0.014**
Locality: cold days				5	3.0	**0.020**

We found that with increasing cold duration, the population‐specific synchrony in emergence increased. The variance of both the post‐winter development time (*t*_*P*_) and the post‐diapause development time (*t*_*D*_) decreased with increasing time in cold (Table S2, Fligner–Killeen test: *t*_*P*_, $\chi_{\mathrm{df}=11}^{2}$ = 56.8, *P* < 0.001; *t*_*D*_, $\chi_{\mathrm{df}=11}^{2}$ = 29.3 *P* = 0.002).

### Weight loss and respiration dynamics during post‐winter development

**Figure 4:**
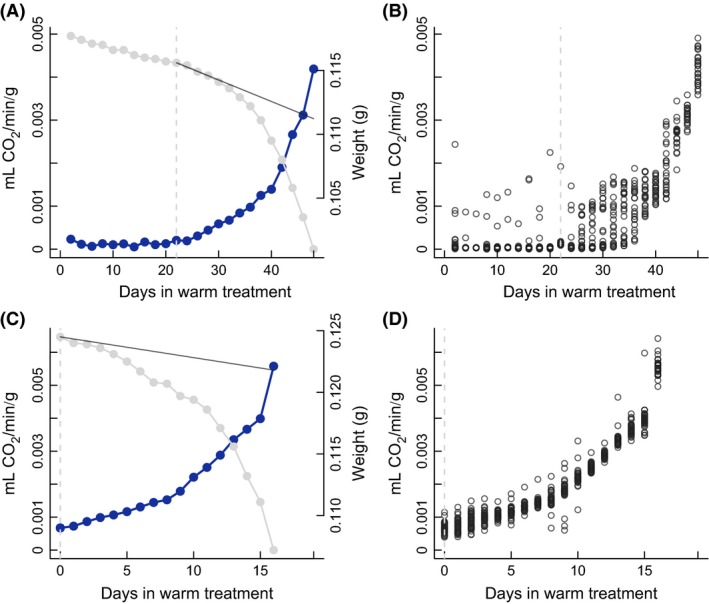
Example of weight loss and respiration of an individual from the 60‐day cold treatment (top row) and an individual from the 150‐day cold treatment (bottom row). (A, C) Weight (gray) and mass corrected CO_2_ production (blue) from day of introduction to warm treatment until hatching. (B, D) Mass corrected CO_2_ production per 45 min cycle. 22–24 cycles were measured overnight. Here, each cycle is represented as a point on the graph. Dashed vertical lines show estimation of termination of diapause based on weight loss. Solid gray line shows cut‐off weight loss slope for diapause termination.

With sufficient time in cold conditions, there was a two‐phase weight loss pattern (Fig. 4C), in agreement with previous results (Stålhandske et al. 2014). However, after shorter cold durations, the separation of diapause and post‐diapause development was not distinct and the interpretation of the weight loss pattern as having clearly separated phases is more problematic. What might in reality be a three‐phase process (diapause and two phases of post‐diapause development) only shows two more‐or‐less distinct weight loss phases (Fig. 4A). Furthermore, the respiration data do not show a clear division into two phases of post‐diapause development, but instead, a pattern of exponentially increasing respiration (Fig. 4). Nevertheless, our criterion for the termination of diapause, based on the rate of weight loss, agrees rather well with the respirometry data, such that the end of diapause as defined by weight loss coincides with the point in time when CO_2_ production starts to increase exponentially (Figs. 4, S1, S2).

### Water content in the two phases of weight loss

Averaging 27% of fresh weight, the water content did not differ between the resting stage and the early and late post‐diapause stages (*F*_2,16_ = 1.03, *P* = 0.38). Thus, the proportion of water remains constant throughout post‐diapause development unlike in some other lepidopterans where post‐diapause weight loss predominantly is due to water loss (Williams et al. 2012). In *A. cardamines,* post‐diapause weight loss therefore likely reflects the use of stored energy to fuel ontogenetic growth.

## Discussion

We found that the duration of cold conditions affected several aspects of post‐winter development in *A. cardamines*. Firstly, the species requires cold in order to break diapause. Secondly, the overall post‐winter development time (*t*_*P*_) of the pupae decreased with increasing cold duration (Fig. 3A and D, Table 1). The effect was a combination of a higher propensity to initiate development immediately, a shorter time until leaving diapause (*t*_0_) for pupae that did not start development immediately, and a shorter post‐diapause development time (*t*_*D*_) for pupae with longer cold duration (Tables 2, 3, 4, Fig. 3). We used the rate of weight loss of individual pupae to determine when they left diapause, and we verified the method using respirometry on a subset of individuals (Figs. 4, S1, S2).

### Pattern of respiration and weight loss during post‐winter development

We found a pattern of increasing respiration rate over the period of post‐winter development. The pattern is similar to what has previously been observed for the European corn borer (Wadsworth et al. 2013) and the apple maggot fly (Ragland et al. 2011). We used a stop‐flow system to measure CO_2_ production over the course of a night, recording the amount of CO_2_ expelled over the duration of one cycle, which was 45 min for individuals from the 30 to 120 days treatment, and 25 min for individuals from the 150 days treatment. Thus, for each individual, there were around 22–24 data points recorded per night. As most cycles returned very little CO_2_, but once or twice during a night a significant CO_2_ amount was measured, respiration in *A. cardamines* likely is through discontinuous gas exchange cycles (DGC) while in diapause. DGCs are commonly seen in diapausing insects and are suggested to carry benefits in terms of water retention, protection against oxidative stress, and pathogen avoidance (Guppy and Withers 1999; Lehmann et al. 2015). After diapause is broken, respiration increases gradually, with DGCs increasing in frequency, approaching a state of continuous gas exchange where spiracles are continuously kept open (Guppy and Withers 1999). In the later part of the post‐diapause development, there is only continuous breathing, at an accelerating rate (Fig. 4B). Overall, these results emphasize the gradualness of the transition from diapause to development initiation in *A. cardamines*. As an increasing number of cycles with significant CO_2_ production coincided with an increase in weight loss (Figs. 4, S1, S2), our method of partitioning diapause and post‐diapause development appears appropriate. CO_2_ production is more directly linked to metabolic rate and development, and will therefore give a higher resolution description of the development from diapause to eclosion. For individuals that experienced sufficient chilling to initiate development immediately when introduced to warm conditions, breathing was continuous from the start (Figs. 4D, S1).

### Differences between localities

Winter conditions differ substantially among the populations we studied (Fig. 2). Applying a method used to estimate chilling hours in fruit trees (viz., hours spent at temperatures below 7.2°C (Luedeling 2015)) to the localities in our study results in a mean difference of over 30 days in chilling duration between southern UK and northern Sweden, when averaged over the years 1998–2012. The mean amount of chilling in Bournemouth, defined in this way, is below 90 days, whereas for northern Sweden it is above 120 days.

A striking result from our experiment is that the southern UK population had noticeably lower chilling requirements than the other populations, and this might be an adaptation to mild winters in the southern UK. The effect we observed was a combination of less chilling needed to break diapause together with a shorter time in diapause as a function of cold duration (Fig. 3B and E). The post‐diapause development time of the southern UK population, on the other hand, was similar to that found in the other populations (Fig. 3C and F).

Masaki (1999) suggested that, for species with constant voltinism, the intensity of diapause should be higher at lower latitudes, as a mechanism to decrease the likelihood of breaking diapause too early. This could apply to *A. cardamines* in Sweden. Individuals from northern Sweden most likely experience sufficiently long winters to approach an upper asymptote in development rate most years, so that selection on diapause duration could be relaxed. In southern Sweden, however, there might be a risk of starting development too soon and the individuals may therefore have a stronger internal control of diapause duration.

### Post‐winter development and spring phenology

For insects at high latitudes, it is common that winter chilling serves to synchronize the life cycle (Hodek and Hodková 1988). The effect of chilling is both an increased developmental sensitivity to increasing spring temperatures and an increased synchrony in hatching for a given spring temperature (e.g., Xiao et al. 2013; Chen et al. 2014; Stålhandske et al. 2014). From our results, this general pattern holds also for *A. cardamines*. A consequence is that, when comparing spring phenology between different localities, one may need to take both differences in winter chilling and in spring warmth into account. Thus, differences between localities in the temperature profile, from late autumn to early summer, need to be considered when evaluating differences in phenology.

Previous studies have found a correlation between *A. cardamines* phenology and spring temperatures (Sparks and Yates 1997; Phillimore et al. 2012; Posledovich et al. 2014; Stålhandske et al. 2014), but the studies that have looked at winter temperatures did not find any effect (Sparks and Yates 1997; Posledovich et al. 2015). However, Posledovich et al. (2015) found an effect of winter duration for Swedish *A. cardamines*, in the form of an increased post‐winter development rate (*r*_*P*_) between 90 and 150 days of winter duration treatment. Here, we have focused on shorter winter durations, from 30 to 120 days, which should be ecologically relevant for UK populations, and we have compared UK and Swedish populations. Because of the relatively short duration of cold in southern UK, it is possible that *A. cardamines* in this region does not experience enough chilling to reach maximal developmental sensitivity to spring warmth, even after taking into account the lower chilling requirements of the southern UK population.

Using field data on adult emergence of *A. cardamines* in the UK, Phillimore et al. (2012) detected a countergradient pattern. They found a latitudinal gradient with the northern UK population appearing to respond more strongly to spring warmth. However, in an experiment where all populations were exposed to 150 days of cold conditions, Stålhandske et al. (2014) found no simple latitudinal pattern, although the southern UK population had a higher post‐winter development rate than the northern one. In our study, post‐winter development times (*t*_*P*_) were similar for the southern and northern UK populations, when controlling for cold duration (a linear model with locality, cold duration, and sex as variable gives a tendency, *P *=* *0.08, toward a shorter time in southern UK). On the other hand, if one compares for a shorter cold duration in southern UK and a longer one in northern UK (e.g., 60 days for southern UK vs. 90 days for northern UK, or 90 days for southern UK vs. 120 days for northern UK; Fig. 3A and D), one sees that, given the winter durations occurring in the field, it is possible that the post‐winter development time as a function of spring temperature would be longer in southern than in northern UK populations. Thus, the seeming discrepancy in geographic patterns of phenology between Phillimore et al. (2012) and Stålhandske et al. (2014) could be the result of geographic differences in winter chilling. Both these studies focused on spring temperatures, and did not take into account that the short winter durations in southern UK might cause a delay in phenology large enough to give rise to a geographic pattern in the field that appears to be countergradient. More generally, the statistical method used to detect local adaptation by Phillimore et al. (2012), and also by Roy et al. (2015), allows for the time of emergence to covary with only one environmental variable (e.g., spring temperature), whereas the combined experimental results of this study and Stålhandske et al. (2014) show that multiple factors can affect the time of emergence.

A further illustration of effects of winter chilling is our finding that British *A. cardamines* have a shorter post‐winter development time for shorter winter durations compared to Swedish individuals (Fig. 3A and D). For longer winters, the difference between the countries is reduced, and after sufficiently long winters, Swedish pupae develop faster than the British, as found by Stålhandske et al. (2014). Because there is little information on how *A. cardamines* pupae accumulate chilling under variable temperature conditions, the nature of geographic variation in the rate of post‐winter development in the field remains an open question. It would be of interest to conduct field experiments, comparing the characteristics of different populations when experiencing field conditions at different localities. This kind of information has long been available for plants, for instance for peach (Fishman et al. 1987).

A warming climate has the potential to either speed up insect post‐winter development, through warmer spring temperatures, or to slow it down, through reduction in winter chilling (Bosch and Kemp 2003; Stålhandske et al. 2014). In North America, it is the minimum temperatures during January and March that have increased the most with climate change (Robeson 2004; ICCP I 2014) indicating that the effects on phenology may be a combination of effects of changes in average winter and spring temperatures. Such combined effects have been investigated in trees and other plants, for which warmer winters slow down development while warmer springs speed up development (Cook et al. 2012; Laube et al. 2014). The matter was discussed in a climate change context for fifteen wild tree species in Britain already in 1989 (Murray et al. 1989), and has since been noted in field studies (Yu et al. 2010) as well as in modeling (Morin et al. 2009). Our study shows that such effects might occur in insects already at present winter temperatures, in that *A. cardamines* individuals in southern UK may be experiencing slowed down post‐winter development due to short winter durations. With even shorter winters, the cold duration might not be sufficient to break diapause after a single winter. It is worth noting that records from the UK report *A. cardamines* passing through two winters in the pupal state (Mitchell 1896), but it is not known whether this occurs in the field.

To summarize, effects of winter condition may be insufficiently recognized in many phenological studies. We found that winter duration affected *A. cardamines* spring phenology through population‐specific effects on diapause duration as well as post‐diapause development. Thus, the possibility of population‐specific responses to winter duration needs to be taken into consideration in order to generate accurate predictions of spring phenology in this and other insects, a task of increasing importance given current climate change scenarios of changing winter conditions.

## Data Accessibility

Data is deposited at Dryad http://dx.doi.org/10.5061/dryad.742fp

## Conflict of Interest

The authors have no conflict of interests to report.

## Supporting information


**Figure S1.** Weight loss and respiration of five individuals from the 60‐day cold treatment and one individual from the 90‐day cold treatment.
**Figure S2.** Proportions of respirometer cycles during which significant CO_2_ production was measured, indicating gas discharge events.
**Table S1.** Number of individuals from each locality in each treatment.
**Table S2.** Standard deviations (days) of the residuals of statistical models of post‐winter (*t*_*p*_) and post‐diapause (*t*_*D*_) development time, with population and sex as explanatory variables.
