# Supplementary material for: Effect of winter cold duration on spring phenology of the orange tip butterfly, Anthocharis cardamines
Source: Ecol Evol. 2015 Nov 7;5(23):5509–20. doi: 10.1002/ece3.1773 (PMC4813107; doi:10.1002/ece3.1773)
Supplement: Supplementary file 1 — Figure S1. Weight loss and respiration of five individuals from the 60‐day cold treatment and one individual from the 90‐day cold treatment. Figure S2. Proportions of respirometer cycles during which significant CO2 production was measured, indicating gas discharge events. Table S1. Number of individuals from each locality in each treatment. Table S2. Standard deviations (days) of the residuals of statistical models of post‐winter (t p) and post‐diapause (t D) development time, with population and sex as explanatory variables. [file ECE3-5-5509-s001.docx]

Supplementary information 1

### Figures

Figure S1. Weight loss and respiration of five individuals from the 60-day cold treatment and one individual from the 90-day cold treatment. Weight (blue) and mass corrected respiration rate (grey) for are shown from day of introduction to warm treatment until hatching. Each dot of mass corrected respiration rate is amount of CO_2_ per 45 min cycle. 22-24 cycles were measured over night. Dashed vertical lines show estimation of termination of diapause based on weight loss. The solid grey line shows the weight loss slope determined to be the daily weight change below which diapause has terminated. The line is shown from the day where diapause is terminated according to this definition.

Figure S2. Proportions of respirometer cycles during which significant CO_2_ production was measured, indicating gas discharge events. A value of 1 indicates continuous gas exchange. The curves show examples of four individuals and the dashed lines reflect the start of development as defined by weight loss (see Methods section).

### Tables

Table S1. Number of individuals from each locality in each treatment

| Cold duration | 30 | 60 | 90 | 120 |
| --- | --- | --- | --- | --- |
| N. Swe | 15 | 17 | 17 | 15 |
| S. UK | 12 | 12 | 13 | 11 |
| N. UK | 10 | 11 | 10 | 10 |
| S . Swe | 6 | 7 | 7 | 6 |

Table S2. Standard deviations (days) of the residuals of statistical models of post-winter (*t_p_*) and post-diapause (*t_D_*) development time, with population and sex as explanatory variables

| **Cold duration** | **N. Sweden** | | **S. UK** | | | **N .UK** | | | | **S. Sweden** | | | |
| --- | --- | --- | --- | --- | --- | --- | --- | --- | --- | --- | --- | --- | --- |
|  | ***t_P_*** | ***t_D_*** | ***t_P_*** | ***t_D_*** | | ***t_P_*** | | ***t_D_*** | | ***t_P_*** | | ***t_D_*** | |
| 60 | 12.2 | 7.3 | 9.8 | 6.6 | 14.4 | | 2.3 | | 7.0 | | 7.0 | |  |
| 90 | 4.7 | 1.9 | 2.1 | 0.9 | 4.3 | | 3.1 | | 4.2 | | 3.2 | |  |
| 120 | 1.6 | 1.4 | 1.9 | 1.5 | 1.6 | | 1.4 | | 2.3 | | 2.1 | | |
